# mHealth Interventions to Promote HIV Self-Testing Among Key Populations: A Systematic Review of Effectiveness and Implementation Outcomes

**DOI:** 10.1177/23259582261431644

**Published:** 2026-04-09

**Authors:** Jann Angeli Agapito, Kyleen Ysabelle Obmerga, Mary Jeanne Pacheco, Catherine Ranin, Amber Sophus, Rogie Royce Carandang

**Affiliations:** 1 College of Pharmacy, 63144 Adamson University, Manila, Philippines; 2 Department of Health, Behavior, and Society, 14742 Kate Marmion School of Public Health, The University of Texas at San Antonio, San Antonio, USA

**Keywords:** HIV, HIV self-testing, key populations, mHealth, mobile health

## Abstract

**Objectives:**

This systematic review synthesized evidence on the effectiveness and implementation outcomes of mobile health (mHealth) interventions supporting HIV self-testing (HIVST) among key populations, including uptake, linkage to care, and user experiences.

**Methods:**

Eight electronic databases and gray literature were searched. Eligible studies evaluated mHealth interventions designed to promote or support HIVST. Due to substantial methodological and contextual heterogeneity, findings were synthesized narratively.

**Results:**

Twenty-one studies from high-, middle-, and low-income settings were included. Interventions included SMS reminders, mobile applications, tele-counseling, and digital outreach platforms. Many studies reported increased HIVST uptake and high acceptability; however, evidence for linkage to care was limited. Study quality varied, with several trials at high risk of bias. Technical barriers, privacy concerns, and challenges in interpreting results were commonly reported.

**Conclusion:**

mHealth interventions appear promising for supporting HIVST among key populations, but current evidence remains limited and preliminary. More rigorous and long-term studies are needed.

## Plain Language Summary Title

mHealth interventions to promote HIV self-testing among key populations

Plain Language Summary

This study reviewed research on how mobile health (mHealth) tools—such as mobile apps, text messages, and online counseling—may support HIV self-testing among people at higher risk of HIV. These groups often face stigma, discrimination, or other barriers that make clinic-based testing difficult. We identified 21 studies from different countries that evaluated digital tools designed to promote or support HIV self-testing. Many studies reported encouraging findings, including increased use of self-testing and high user satisfaction. However, the overall quality of the evidence varied, and several studies had important methodological limitations. Only one study examined whether digital support improved connection to confirmatory testing and care, and very few assessed longer-term outcomes such as sustained engagement, cost, or scalability. Because of differences in study design and quality, the findings should be interpreted with caution. Overall, mHealth approaches appear promising as a way to expand access to HIV self-testing. However, current evidence remains limited and preliminary. More rigorous and long-term research is needed to better understand their effectiveness and how they can be successfully implemented across different populations and settings.

## Introduction

The global HIV epidemic remains a pressing public health challenge, with an estimated 40 million people living with HIV as of 2023.^1^ Key populations that bear a disproportionate burden of HIV infection include men who have sex with men (MSM), female sex workers (FSWs), people who inject drugs (PWIDs), transgender individuals, and incarcerated persons.^2^ The persistence of the epidemic is driven by a complex interplay of behavioral, structural, and social determinants—including systemic inequities, social marginalization, HIV-related stigma and discrimination, and limited access to healthcare services (eg, lack of health insurance).^3^

HIV testing serves as the critical entry point to the HIV care cascade, linking individuals to prevention, diagnosis, and treatment services. However, despite decades of global efforts, substantial gaps in testing coverage remain. As of 2023, only 86% of people living with HIV were aware of their status, underscoring the urgent need to expand testing access.^4^ Limited uptake of HIV testing continues to delay diagnosis and treatment initiation, impeding progress toward global prevention goals. While traditional strategies—such as condom promotion, provider-initiated testing, and facility-based counseling—have demonstrated effectiveness,^5^ they often fail to reach hidden, underserved, or reluctant populations who avoid formal healthcare systems due to stigma, confidentiality concerns, or logistical barriers. Expanding discreet, user-centered approaches to HIV testing is therefore essential to narrowing the testing gap and achieving the UNAIDS 95–95–95 targets.^6^

HIV self-testing (HIVST), recommended by the World Health Organization (WHO), has emerged as a promising and innovative strategy to overcome these challenges.^7^ HIVST is a process in which individuals collect their own blood or oral fluid sample, conduct the diagnostic test, and interpret their results independently.^8^ By allowing users to test in private, HIVST addresses barriers related to stigma, confidentiality, and clinic accessibility.^5^ Evidence from global trials and systematic reviews indicates that HIVST is acceptable, effective, and cost-saving across diverse populations, often demonstrating uptake rates comparable to or exceeding those of conventional testing methods, particularly among individuals hesitant to visit health facilities.^9,10^ However, despite its promise, the potential of HIVST remains constrained by user errors, limited post-test counseling, and poor linkage to confirmatory testing and care.^11^ Strengthening linkage pathways following a reactive result remains a critical yet underdeveloped aspect of the HIVST cascade.

In this context, mobile health (mHealth) interventions—including SMS reminders, mobile applications, chatbots, and digital counseling platforms—may offer a scalable, adaptable, and cost-effective means of enhancing HIV prevention and self-testing initiatives. mHealth tools have demonstrated significant impacts on antiretroviral therapy (ART) adherence, appointment attendance, and improvement in HIV-related knowledge.^12,13^ These platforms also facilitate test kit ordering, educational outreach, and real-time virtual support, directly addressing barriers associated with traditional testing modalities.

Yet, despite growing enthusiasm, evidence regarding the effectiveness of mHealth interventions tailored specifically for HIVST and linkage to care among key populations remains limited. Much of the existing literature focuses on general HIV prevention or ART adherence, leaving critical gaps in understanding how digital tools can enhance self-testing uptake and continuity of care. Moreover, little is known about the implementation outcomes—such as feasibility, acceptability, and user satisfaction—of mHealth-enabled HIVST programs, especially among vulnerable or marginalized groups.

This systematic review seeks to fill this evidence gap by synthesizing current literature on mHealth interventions designed to promote HIVST and facilitate linkage to care among key populations. By examining both effectiveness and implementation outcomes, the review aims to inform public health strategies, guide the design of digital HIV programs, and support evidence-based policy recommendations to optimize testing access through mHealth innovations.

## Methods

### Study Design and Registration

This systematic review was conducted in accordance with the Preferred Reporting Items for Systematic Reviews and Meta-Analyses (PRISMA) 2020 guidelines (Supplementary File 1). The review protocol was registered with the International Prospective Register of Systematic Reviews (PROSPERO) under the registration number CRD42023479209 (Supplementary File 2). The review was conducted between January 1, 2024, and February 28, 2025.

### Eligibility Criteria

#### Population

The review focused on studies involving key populations at increased risk of HIV infection. These comprised FSWs, MSM, gay men, transgender individuals, PWIDs, heterosexuals, pregnant women, adolescents, and incarcerated individuals.

#### Intervention

Eligible studies evaluated mHealth interventions specifically designed to support or promote HIVST. The characteristics and functionalities of these interventions were conceptualized using the mHealth and Information and Communication Technology (ICT) Framework, which provides a foundation for understanding how digital tools facilitate health research, communication, and service delivery.^14^

Included interventions encompassed a range of mobile phone-based technologies, such as SMS reminders, mobile applications, telemedicine consultations, and chat-based digital counseling platforms. Studies utilizing digital platforms that did not involve mobile or wireless technologies (eg, desktop-only systems or offline software) were excluded from the review.

#### Comparator

Comparators included standard HIV testing services, usual care, or facility-based testing. Studies without explicit comparators were also included if they presented pre- and post-intervention data or reported relevant qualitative findings.

#### Outcomes

To avoid conceptual overlap, effectiveness outcomes were defined as participant-level behavioral or clinical impacts of mHealth-supported HIVST, whereas implementation outcomes referred to the feasibility, appropriateness, and delivery-related aspects of the intervention. The primary outcomes of this review focused on the effectiveness of mHealth interventions in promoting HIVST among key populations. These included 1) HIVST uptake: the proportion of individuals who completed a self-test following exposure to an mHealth intervention^15^; 2) willingness to continue testing: measured through self-reported intentions or behaviors indicating repeat testing^16^; 3) linkage to care: the proportion of individuals with reactive test results who accessed confirmatory testing and were referred to care^17^; and 4) user satisfaction: participants’ perceptions of the intervention's usefulness, relevance, and overall experience.^18^

The secondary outcomes pertain to implementation aspects based on Proctor et al's implementation outcomes framework.^19^ These included 1) acceptability: the perceived appropriateness and comfort with using the mHealth platform and HIVST process; and 2) implementation barriers: concerns about privacy, difficulties with usability, and contextual challenges like digital literacy or access to technology. The review also incorporates recommendations from the included studies to inform the future design and implementation of mHealth interventions.

In this review, satisfaction was conceptualized as an effectiveness outcome reflecting perceived benefit of the HIVST service experience, whereas acceptability was conceptualized as an implementation outcome reflecting intervention feasibility and delivery. These constructs were analyzed and reported separately to prevent conceptual duplication.

#### Setting and Context

This review had a global scope, with no geographic restrictions. Only English-language articles published between December 1, 2016, and January 1, 2024, were included, corresponding to the period following the WHO's recommendation of HIVST in 2016.^20^

#### Information Sources and Search Strategy

A comprehensive search was conducted across eight electronic databases: PubMed/Medical Literature Analysis and Retrieval System Online (MEDLINE), Web of Science, Cumulative Index to Nursing and Allied Health Literature (CINAHL), Academic Search Complete, PsycArticles, Psychological Information Database (PsycINFO), Sociological Index (SocINDEX), and the Cochrane Central Register of Controlled Trials (CENTRAL). Gray literature was sourced from relevant organizations, including the WHO, the United States Centers for Disease Control and Prevention (CDC), the European Centre for Disease Prevention and Control (ECDC), the United Nations Children's Fund (UNICEF), and the Joint United Nations Programme on HIV/AIDS (UNAIDS).

Search terms and Boolean operators were used to identify studies related to key populations, HIVST, and mHealth. Controlled vocabulary (eg, Medical Subject Headings [MeSH] terms) and keyword strategies were tailored to the indexing structure of each database. The full search strategies are available in (Supplementary File 3).

#### Study Selection and Data Extraction

Two reviewers independently screened the titles, abstracts, and full texts of identified records against predefined eligibility criteria. Disagreements were resolved through discussion or by consulting a third reviewer. A structured data extraction form was used to collect key information, including study identifiers, design, country, population characteristics, sample size, intervention details, outcomes, challenges, and recommendations (Supplementary File 4).

#### Types of Studies to be Included

The review included a broad range of study designs, including randomized controlled trials (RCTs), quasi-experimental studies, cohort and case-control studies, cross-sectional studies, mixed-methods studies, qualitative studies, and implementation evaluations. Studies that did not present original data (ie, protocols, editorials, commentaries, letters, reviews, conference abstracts, and books) were excluded.

#### Quality Appraisal and Risk of Bias Assessment

Each included study was assessed for methodological quality using tools appropriate to its study design. For RCTs, the Cochrane Risk of Bias 2.0 (RoB 2) tool was used.^21^ Quasi-experimental studies were evaluated using the Risk Of Bias In Non-randomized Studies of Interventions (ROBINS-I) tool,^22^ while observational studies were appraised with the National Institutes of Health Quality Assessment Tool.^23^ The Critical Appraisal Skills Programme checklist was applied to qualitative studies,^24^ and the Mixed Methods Appraisal Tool was used for mixed-methods studies.^25^

The narrative weight of evidence technique was used to sum up the findings from various quality rating systems instead of quantitative aggregation. Study quality and risk of bias assessments were used to guide how much confidence we placed in individual findings and how strongly they informed the overall synthesis and recommendations. No study was disqualified based on the strength of its methodology.

To assess the certainty of the evidence, the Grading of Recommendations Assessment, Development and Evaluation (GRADE) framework was applied for quantitative outcomes.^26,27^ This evaluation considered domains such as risk of bias, consistency, directness, precision, and publication bias. For qualitative findings, the GRADE-CERQual (Confidence in the Evidence from Reviews of Qualitative research) approach was used to assess confidence based on methodological rigor, coherence, adequacy of data, and relevance to the review question.^28^ Certainty of evidence using GRADE for quantitative outcomes and GRADE-CERQual for qualitative findings paralleled appraisal results, and thus conclusions were done cautiously where the majority of evidence came from studies with a higher risk of bias.

#### Data Synthesis and Analysis

Given substantial heterogeneity in study designs, intervention modalities, outcome definitions, and measurement approaches, meta-analysis was not appropriate. We therefore conducted a structured narrative synthesis in accordance with the Synthesis Without Meta-analysis reporting guidelines (Supplementary File 5). Quantitative studies were synthesized by examining the direction and magnitude of associations between mHealth interventions and key outcomes, such as HIVST uptake and linkage to care. Qualitative studies were synthesized using an inductive thematic approach that emphasized patterns and meanings derived from participant narratives and reported themes. As specified in the PROSPERO protocol, initially subgroup analysis was planned if enough RCTs were available. However, due to the limited number of RCTs, subgroup analysis was precluded. This deviation from the protocol was reported in accordance with PRISMA guidelines.

Quantitative and qualitative findings were integrated to provide a comprehensive interpretation of effectiveness and implementation outcomes. Findings were categorized into three groups based on reported effects: positive, no effect, or mixed. Results were classified as positive if mHealth interventions improved outcomes such as HIVST uptake, user satisfaction, willingness to test, or linkage to care; no effect if the intervention produced minimal or no measurable impact; and mixed if results were inconsistent or constrained by implementation challenges that limited effectiveness.

Certainty of evidence was considered using the GRADE framework for quantitative findings and GRADE-CERQual for qualitative evidence. Findings were iteratively reviewed to ensure consistency in interpretation and to capture variations across populations, geographic contexts, and mHealth modalities. This approach enabled a nuanced, context-sensitive synthesis while yielding clear, actionable insights into how mHealth interventions shape HIVST behaviors and service engagement among key populations.

## Results

### Study Selection

**Figure 1.**
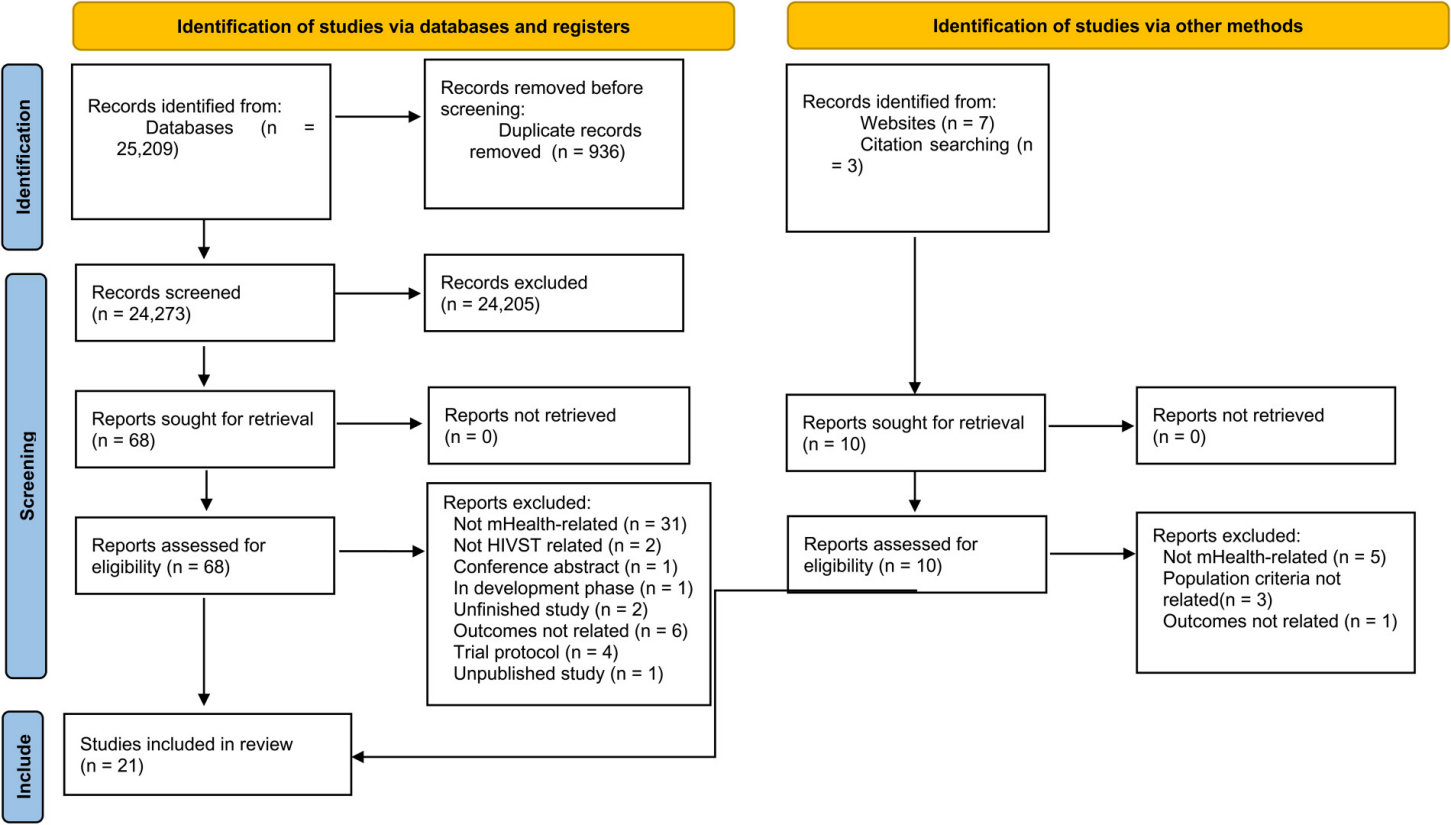
PRISMA 2020 flow diagram of the article selection process. PRISMA, Preferred Reporting Items for Systematic Reviews and Meta-Analyses.

A total of 25 209 records were identified through database searches, ten from gray literature sources, and three from manual reference checks. After removing duplicates, 24 273 records remained. Based on title and abstract screening, 68 articles were selected for full-text review. Following a thorough eligibility screening, 21 studies were included for this review. The complete selection process is detailed in the PRISMA flow diagram (Figure 1), while excluded studies with reasons are provided in Supplementary File 6.

### Risk of Bias and Methodological Quality

**Figure 2.**
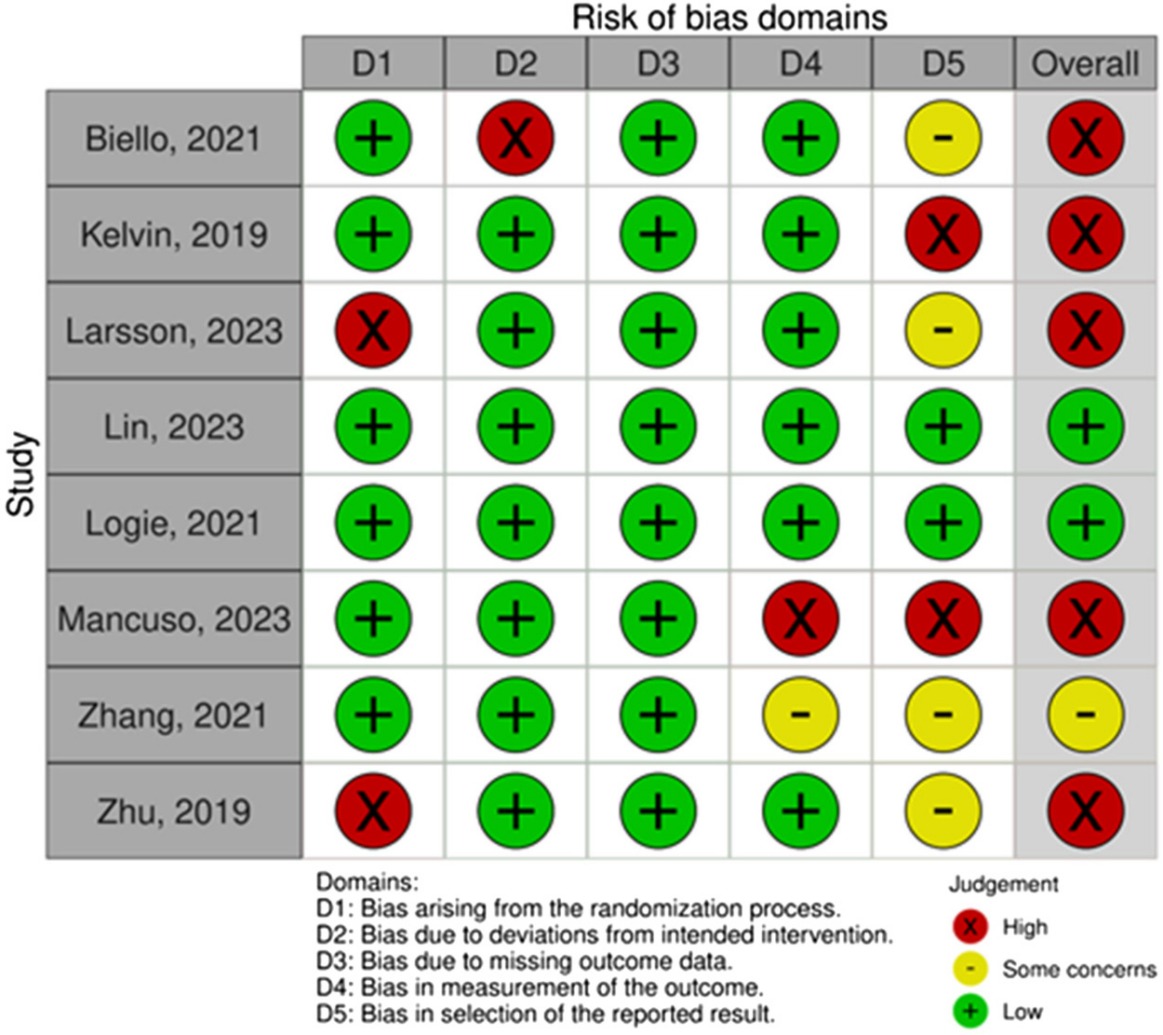
Risk of bias summary of randomized controlled trials based on authors’ judgement **(N** **=** **8)**.

The risk of bias and methodological rigor varied across the included studies (Supplementary File 7). Among the eight RCTs, five were assessed as having a high risk of bias due to shortcomings in randomization procedures, lack of blinding, and selective reporting. Two RCTs were considered low risk, while one had some concerns (Figure 2). One quasi-experimental study showed a moderate risk of bias. Among the eight observational studies, most lacked clarity in reporting sample size justification, power calculations, and variance estimations. Three mixed-methods studies failed to report confounding variables, and one qualitative study demonstrated high methodological quality. The certainty of evidence, as assessed through GRADE for quantitative studies and GRADE-CERQual for qualitative findings, is summarized in Supplementary File 8 and Supplementary File 9.

### Study Characteristics

The 21 included studies varied in design: eight were RCTs, six cross-sectional studies, two cohort studies, three mixed-methods studies, one quasi-experimental, and one qualitative study. Most studies evaluated the effects of mHealth interventions on multiple outcomes, with the majority reporting positive findings related to HIVST.

### Geographic Distribution

The studies were geographically diverse. Based on 2023 World Bank income classifications,^29^ more than half (57%) were conducted in upper-middle-income countries: China (n = 7), South Africa (n = 3), Brazil (n = 1), and Malaysia (n = 1). Five studies (24%) were conducted in a high-income country, all of which were conducted in the United States. Three studies (14%) were conducted in lower-middle-income countries—two in Kenya and one in Zimbabwe. Only one study (5%) was conducted in a low-income country, Uganda.

### Study Populations

Twelve studies (57%) focused on MSM, reflecting both epidemiologic vulnerability and programmatic interest. Four studies recruited general adult populations aged 18 and above, provided they had access to smartphones. Three studies each targeted cisgender men and youth aged 16-24, while one study focused specifically on FSWs, and another study focused on HIV-negative women aged 14 and older.

### Types of mHealth Interventions

The studies employed a wide range of mHealth modalities. The most common intervention type was mobile applications (n = 13, 62%). Three studies employed SMS-based interventions, and one study used a chatbot. Additional modalities included smartphone-based electronic readers, social media campaigns, and advertisements through geosocial networking apps like Grindr™. Using the mHealth and ICT Framework, intervention functionalities were categorized into six domains: health education or promotion, self-monitoring, laboratory result submission, reminders, HIV care support, and data collection/reporting. Many studies integrated multiple functionalities, highlighting the layered nature of mHealth systems.

### Effectiveness Outcomes

The effectiveness of study outcomes was organized into four categories: HIVST uptake, willingness to continue testing, linkage to care, and user satisfaction (Table 1).

**Table 1.** Summary of Effectiveness and Implementation Strategies of mHealh Interventions on HIVST.

Outcomes	Types of MHealth Services (n)	No Effect (n)	Mixed Effect (n)	Positive Effect (%)	Total Number of Outcomes Reported
Effectiveness					
HIVST uptake ^13,30–42^	Advertisements (1) App (10) Online via phone (1) SMS (2)	1	0	13 (93)	14
Willingness to continue using HIVST ^35,36,38,42,43^	App (4) SERs (1)	0	0	5 (100)	5
Linkage to care ^44^	App (1)	0	0	1 (100)	1
User satisfaction ^45^	Online via phone (1)	0	0	1 (100)	1
Implementation					
mHealth intervention acceptability ^30,32,45–47^	App (3) Chatbot (1) Online via phone (1)	0	0	5 (100)	5
Acceptability of the HIVST process ^33,48,49^	Advertisement (1) App (1) SMS (1)	0	0	3 (100)	3
Implementation barriers and recommendations^13,30,33,34,36,43–41,42,46–49^	Advertisement (1) App (10) Chatbot (1) SERs (1) SMS (2)	-	-	-	15

*Notes.* App, application; SERs, smartphone-based electronic readers; SMS, short message service; mHealth, mobile health; HIVST, HIV self-testing.

### HIVST Uptake

Fourteen studies assessed HIVST uptake, defined as the proportion of participants who used an HIVST following mHealth exposure. Of these, 13 studies reported improved uptake, while one study found no significant effect. For example, the SMARTtest app achieved a 77% uptake rate, with an average of 3.7 test uses per participant.^30^ The MyChoices and LYNX apps facilitated distribution of over 2500 HIVST kits, while the Aspect™ app showed an 89% completion rate among users.^32–34^ Social media-linked interventions also demonstrated strong uptake: Grindr™-based campaigns generated 4389 unique website visits and 333 test requests during a single 4-week period. Other platforms like WeTest and JomPrEP demonstrated similar engagement levels, especially when supported by real-time user interaction or peer facilitation.^35,50^ Overall, the CERQual certainty for this outcome was rated as low, primarily due to study heterogeneity and some concerns about implementation fidelity.

### Willingness to Continue Using HIVST

Five studies reported on willingness to continue using HIVST, all showing strong intent to maintain regular self-testing behaviors. In China, a cross-sectional study found that 71.2% of MSM were willing to use a self-test result system, and 98% believed it would increase testing frequency.^36,43^ In Malaysia, the JomPrEP app saw 84% of participants express intention to continue use, with similarly high satisfaction scores.^36^ High willingness was also reported in cohort and qualitative studies conducted in South Africa and China.^35,44^ This outcome had low CERQual certainty due to the limited number of studies but consistent positive findings.

### Linkage to Care

One quasi-experimental study in South Africa evaluated linkage to care, revealing that 99.7% of HIVST users accessed confirmatory testing and were referred for treatment.^45^ This was notably higher than the linkage rate observed in facility-based testing groups. The study also observed a greater number of new HIV diagnoses in the intervention arm, suggesting that mHealth-supported HIVST may be effective for reaching previously undiagnosed individuals. The GRADE certainty for this outcome was rated as very low, reflecting the limited number of studies and non-randomized design.

### User Satisfaction

Only one study reported on satisfaction with mHealth-based HIVST. This mixed-methods study, conducted in Hong Kong, found satisfaction levels ranging from 88.8% to 96.8%, particularly among users who accessed real-time online counseling services.^45^ Participants highlighted emotional reassurance and improved understanding of test results as key benefits. The CERQual confidence in this finding was rated as low, as it was based on a single study.

### Implementation Outcomes

Implementation outcomes were organized by acceptability of the mHealth intervention; acceptability of the HIVST process; and implementation barriers.

### Acceptability of mHealth Intervention

Five studies examined the acceptability of mHealth intervention. Users rated features such as ease of use, functionality, and relevance to their daily lives positively. For example, SMARTtest received a mean usability rating of 4.16 out of 5, while MyChoices and LYNX were deemed extremely helpful by 87% of users.^32,33^ The E-testing app received over 2500 downloads and participants in a study using the Nolwazi chatbot reported feeling as though they were interacting with a real person.^46^ The CERQual certainty for this outcome was moderate, supported by multiple study designs and populations.

### Acceptability of the HIVST Process

Three studies specifically examined the acceptability of the HIVST process delivered through mHealth platforms. All reported favorable findings, indicating that participants generally found the process acceptable and user-friendly. In a Los Angeles-based study using Grindr™, 93% of participants reported ease of use, and 77% preferred self-testing over clinic-based testing.^34,38^ Similar findings emerged from studies in China and Kenya, where acceptability was enhanced through social media platforms and SMS reminders.^39,51^ The GRADE certainty for this outcome was moderate, supported by consistent evidence across diverse settings.

### Implementation Barriers and Study Recommendations

Common barriers to implementation included user-related challenges, such as mistakes in using test kits, difficulty understanding instructions, and technical issues like poor internet connectivity, low digital literacy, and privacy concerns. Structural challenges included limited access to advanced testing kits, especially in low-resource settings, and sociocultural barriers such as HIV-related stigma and discrimination. To address these barriers, several studies recommended improving kit usability, integrating anonymous testing options, strengthening data security protocols, enhancing digital literacy training, and reducing cost barriers. Additionally, interventions featuring human-like AI (eg, empathetic chatbots) and targeted marketing through social apps were suggested to improve uptake and engagement.

## Discussion

This systematic review highlights the growing potential of mHealth interventions to promote HIVST among key populations. Across diverse geographic and demographic contexts, mHealth platforms—including mobile applications, SMS reminders, chatbots, and online outreach tools—have been shown to increase HIVST uptake, enhance user satisfaction, encourage repeat testing. However, evidence regarding its ability to support linkage to confirmatory testing and care remains limited and uncertain. These interventions offer private, user-centered alternatives to conventional HIV testing, mitigating persistent barriers such as stigma, confidentiality concerns, and limited healthcare access that disproportionately affect marginalized and hard-to-reach populations.

Findings suggest that mHealth interventions may improve the acceptability and usability of HIVST, particularly in low-resource settings; however, the certainty of the evidence ranges from low to moderate, while evidence related to linkage to care is of very low certainty. Participants consistently described these tools as intuitive, convenient, and accessible, often preferring them over facility-based services. Interventions integrating real-time counseling, peer support, or interactive educational features were especially well received.^37,46,48^ Even low-bandwidth modalities—such as SMS reminders—proved effective in maintaining engagement, underscoring their feasibility and scalability in areas with limited internet access but high mobile phone penetration. This review aligns with earlier evidence on the effectiveness of SMS- and app-based interventions while also emphasizing emerging platforms such as chatbots and dating applications. For instance, interventions delivered through Grindr™ and WeChat not only increased testing uptake but also improved perceptions of privacy, convenience, and engagement—factors particularly important for MSM, who often face stigma and discrimination in traditional healthcare systems.^34,35,42,49^

Despite these promising results, several implementation challenges persist. Technical barriers such as unreliable connectivity, limited digital literacy, and usability issues were common, particularly in resource-constrained environments.^45–47^ Concerns about data privacy, confidentiality, and difficulties in interpreting self-test results also emerged as recurrent themes.^36,40,44,45^ Although some quantitative studies reported technical barriers, overall acceptability of mHealth HIVST remained high. Qualitative findings suggest that participants’ appreciation for privacy, convenience, and autonomy helped mitigate these challenges. Together, these insights highlight how user experience and perceived benefits can explain and complement the quantitative outcomes. Moreover, most studies focused on short-term outcomes such as uptake and satisfaction, while few examined long-term behavioral changes, sustained engagement in care, or broader health impacts. Only one study followed participants beyond self-testing to assess confirmatory testing or behavioral outcomes,^41^ and none evaluated cost, scalability, or sustainability. These limitations highlight the need to strengthen the evidence base through more comprehensive evaluations of intervention effectiveness and real-world feasibility.

The success of mHealth initiatives was found to be highly context dependent. In LMICs, implementation requires careful consideration of technological access, cultural relevance, language preferences, and integration within existing health systems. Simple, discreet interventions embedded in platforms already familiar to target populations are more likely to achieve acceptance, scalability, and sustainability.^52^ However, ensuring equity in digital health remains a key challenge, particularly for populations with limited access to mobile technology or stable internet connections.

While this review provides comprehensive evidence on the effectiveness and selected implementation outcomes of mHealth-supported HIVST, important gaps remain. Evidence is fragmented across contexts, with limited assessment of core implementation science outcomes, including fidelity, equity, cost-effectiveness, scalability, and sustainability. Notably, only one study evaluated linkage to confirmatory care, and none systematically assessed cost-effectiveness or long-term sustainability. The lack of longitudinal data further limits understanding of sustained engagement and integration into routine health systems. Additionally, the predominance of studies from high-income countries restricts generalizability to LMICs, where mHealth interventions may have the greatest potential to expand testing access. To advance the field, future research should move beyond short-term acceptability studies and adopt longitudinal, mixed-methods designs to assess sustained HIVST use, linkage to care, and health system integration. Studies should explicitly measure key implementation outcomes—including fidelity, cost, scalability, sustainability, equity, and long-term engagement—to generate policy-relevant evidence for scale-up. Strengthening digital literacy, privacy protections, and data governance frameworks will also be critical to ensure trust and sustained participation, particularly among vulnerable populations such as MSM, sex workers, and transgender individuals.

Based on the evidence synthesized, several practical recommendations can guide the optimization of mHealth-supported HIVST programs. However, the implications for practice extend beyond general calls to improve digital literacy or strengthen collaboration. Greater clarity is needed regarding who should act and at what stage of implementation. In the immediate term, researchers should prioritize the routine measurement of implementation indicators such as fidelity, linkage to care, and intervention costs alongside uptake outcomes, while policymakers should formally integrate mHealth-supported HIVST into national HIV frameworks and ensure regulatory protections for data security and confidentiality. Implementers and community-based organizations should focus on culturally responsive adaptation of interventions and embedding services within platforms already trusted by key populations. Over time, research efforts must shift toward longitudinal evaluation of sustained engagement, cost-effectiveness, scalability, and health system integration, particularly in LMIC contexts. Concurrently, policymakers should establish sustainable financing pathways and strengthen digital infrastructure to support expansion, and implementers should institutionalize monitoring systems that assess long-term engagement and program continuity. Presenting recommendations in this manner enhances their clarity and operational relevance for stakeholders responsible for translating evidence into routine practice. Finally, fostering multi-sectoral collaboration among governments, community-based organizations, and technology partners will strengthen implementation and maximize public health impact.

This review has several limitations. First, heterogeneity in study designs, target populations, and intervention modalities limited comparability and precluded meta-analysis. Second, although most studies reported positive effects of mHealth interventions on HIVST outcomes, these findings should be interpreted with caution. Five of the eight included RCTs were assessed as having a high risk of bias due to limitations in randomization, blinding, and outcome reporting. The predominance of high-risk studies weakens causal inference and may overestimate intervention effects. Therefore, findings should be interpreted with caution. Future research should prioritize more rigorously designed RCTs with improved randomization procedures, appropriate blinding where feasible, and transparent reporting to strengthen the evidence base. Consequently, while mHealth-supported HIVST interventions appear promising, conclusions regarding their effectiveness remain provisional. Third, restricting the review to English-language and mobile-based interventions may have excluded relevant evidence from non-English or non-mobile platforms. Fourth, generalizability was limited by the narrow geographic and population distributions of the included studies. Most were conducted in upper-middle-income countries—primarily China and South Africa—and focused largely on MSM. Although this reflects current programmatic priorities, it restricts applicability to other key populations and settings. Evidence remains scarce for sex workers, transgender persons, PWIDs, and populations in low-income countries, where structural and digital barriers may differ. Future research should prioritize these underrepresented groups to improve the equity and global relevance of mHealth-supported HIVST interventions. Fifth, most studies focused on proximal outcomes such as uptake and acceptability, with limited assessment of longer term implementation outcomes, including cost-effectiveness, fidelity, and sustainability. Addressing these limitations through more rigorous study designs and broader inclusion criteria will strengthen future evidence synthesis and intervention development. Finally, only English-language studies were included, potentially excluding relevant research published in other languages. This language restriction may limit the overall representativeness of the findings. Future updates of this review should consider including non-English studies to provide a more comprehensive assessment of the evidence.

Despite these limitations, this review contributes methodologically by offering the first integrated synthesis of effectiveness and implementation outcomes of mHealth-supported HIVST interventions among key populations. By combining a comprehensive search strategy with established appraisal frameworks such as GRADE and GRADE-CERQual, the review provides a transparent and structured evidence base to guide interpretation and future inquiry.

## Conclusions

This systematic review suggests that mHealth interventions have the potential to support HIVST among key populations. Tools such as mobile applications, SMS reminders, and digital outreach platforms were associated with increased testing uptake, higher user satisfaction, and engagement with HIV prevention by addressing barriers related to stigma, privacy concerns, and limited access to facility-based services. However, these findings should be interpreted with caution given the mixed quality and heterogeneity of the underlying evidence. Their potential impact is further constrained by challenges including technical limitations, gaps in digital literacy, and concerns about data security. In addition, much of the existing evidence focuses on short-term outcomes, with limited assessment of long-term behavior change, sustained linkage to care, or broader implementation outcomes such as cost-effectiveness, scalability, and equity. The predominance of observational studies and small trials highlights the need for larger, well-designed RCTs to better establish the strength of the evidence. Future research should therefore emphasize more rigorous study designs, system-level integration, and equitable implementation to better understand the role of mHealth-supported HIVST in advancing global HIV prevention efforts.

## Supplemental Material

sj-pdf-1-jia-10.1177_23259582261431644 - Supplemental material for mHealth Interventions to Promote HIV Self-Testing Among Key Populations: A Systematic Review of Effectiveness and Implementation Outcomes Supplemental material, sj-pdf-1-jia-10.1177_23259582261431644 for mHealth Interventions to Promote HIV Self-Testing Among Key Populations: A Systematic Review of Effectiveness and Implementation Outcomes by Jann Angeli Agapito, Kyleen Ysabelle Obmerga, Mary Jeanne Pacheco, Catherine Ranin, Amber Sophus and Rogie Royce Carandang in Journal of the International Association of Providers of AIDS Care (JIAPAC)

sj-docx-2-jia-10.1177_23259582261431644 - Supplemental material for mHealth Interventions to Promote HIV Self-Testing Among Key Populations: A Systematic Review of Effectiveness and Implementation Outcomes Supplemental material, sj-docx-2-jia-10.1177_23259582261431644 for mHealth Interventions to Promote HIV Self-Testing Among Key Populations: A Systematic Review of Effectiveness and Implementation Outcomes by Jann Angeli Agapito, Kyleen Ysabelle Obmerga, Mary Jeanne Pacheco, Catherine Ranin, Amber Sophus and Rogie Royce Carandang in Journal of the International Association of Providers of AIDS Care (JIAPAC)

sj-pdf-3-jia-10.1177_23259582261431644 - Supplemental material for mHealth Interventions to Promote HIV Self-Testing Among Key Populations: A Systematic Review of Effectiveness and Implementation Outcomes Supplemental material, sj-pdf-3-jia-10.1177_23259582261431644 for mHealth Interventions to Promote HIV Self-Testing Among Key Populations: A Systematic Review of Effectiveness and Implementation Outcomes by Jann Angeli Agapito, Kyleen Ysabelle Obmerga, Mary Jeanne Pacheco, Catherine Ranin, Amber Sophus and Rogie Royce Carandang in Journal of the International Association of Providers of AIDS Care (JIAPAC)

sj-pdf-4-jia-10.1177_23259582261431644 - Supplemental material for mHealth Interventions to Promote HIV Self-Testing Among Key Populations: A Systematic Review of Effectiveness and Implementation Outcomes Supplemental material, sj-pdf-4-jia-10.1177_23259582261431644 for mHealth Interventions to Promote HIV Self-Testing Among Key Populations: A Systematic Review of Effectiveness and Implementation Outcomes by Jann Angeli Agapito, Kyleen Ysabelle Obmerga, Mary Jeanne Pacheco, Catherine Ranin, Amber Sophus and Rogie Royce Carandang in Journal of the International Association of Providers of AIDS Care (JIAPAC)

sj-pdf-5-jia-10.1177_23259582261431644 - Supplemental material for mHealth Interventions to Promote HIV Self-Testing Among Key Populations: A Systematic Review of Effectiveness and Implementation Outcomes Supplemental material, sj-pdf-5-jia-10.1177_23259582261431644 for mHealth Interventions to Promote HIV Self-Testing Among Key Populations: A Systematic Review of Effectiveness and Implementation Outcomes by Jann Angeli Agapito, Kyleen Ysabelle Obmerga, Mary Jeanne Pacheco, Catherine Ranin, Amber Sophus and Rogie Royce Carandang in Journal of the International Association of Providers of AIDS Care (JIAPAC)

sj-pdf-6-jia-10.1177_23259582261431644 - Supplemental material for mHealth Interventions to Promote HIV Self-Testing Among Key Populations: A Systematic Review of Effectiveness and Implementation Outcomes Supplemental material, sj-pdf-6-jia-10.1177_23259582261431644 for mHealth Interventions to Promote HIV Self-Testing Among Key Populations: A Systematic Review of Effectiveness and Implementation Outcomes by Jann Angeli Agapito, Kyleen Ysabelle Obmerga, Mary Jeanne Pacheco, Catherine Ranin, Amber Sophus and Rogie Royce Carandang in Journal of the International Association of Providers of AIDS Care (JIAPAC)

sj-pdf-7-jia-10.1177_23259582261431644 - Supplemental material for mHealth Interventions to Promote HIV Self-Testing Among Key Populations: A Systematic Review of Effectiveness and Implementation Outcomes Supplemental material, sj-pdf-7-jia-10.1177_23259582261431644 for mHealth Interventions to Promote HIV Self-Testing Among Key Populations: A Systematic Review of Effectiveness and Implementation Outcomes by Jann Angeli Agapito, Kyleen Ysabelle Obmerga, Mary Jeanne Pacheco, Catherine Ranin, Amber Sophus and Rogie Royce Carandang in Journal of the International Association of Providers of AIDS Care (JIAPAC)

sj-pdf-8-jia-10.1177_23259582261431644 - Supplemental material for mHealth Interventions to Promote HIV Self-Testing Among Key Populations: A Systematic Review of Effectiveness and Implementation Outcomes Supplemental material, sj-pdf-8-jia-10.1177_23259582261431644 for mHealth Interventions to Promote HIV Self-Testing Among Key Populations: A Systematic Review of Effectiveness and Implementation Outcomes by Jann Angeli Agapito, Kyleen Ysabelle Obmerga, Mary Jeanne Pacheco, Catherine Ranin, Amber Sophus and Rogie Royce Carandang in Journal of the International Association of Providers of AIDS Care (JIAPAC)

sj-pdf-9-jia-10.1177_23259582261431644 - Supplemental material for mHealth Interventions to Promote HIV Self-Testing Among Key Populations: A Systematic Review of Effectiveness and Implementation Outcomes Supplemental material, sj-pdf-9-jia-10.1177_23259582261431644 for mHealth Interventions to Promote HIV Self-Testing Among Key Populations: A Systematic Review of Effectiveness and Implementation Outcomes by Jann Angeli Agapito, Kyleen Ysabelle Obmerga, Mary Jeanne Pacheco, Catherine Ranin, Amber Sophus and Rogie Royce Carandang in Journal of the International Association of Providers of AIDS Care (JIAPAC)
