# Supplementary material for: mHealth Interventions to Promote HIV Self-Testing Among Key Populations: A Systematic Review of Effectiveness and Implementation Outcomes
Source: J Int Assoc Provid AIDS Care. 2026 Apr 9;25:23259582261431644. doi: 10.1177/23259582261431644 (PMC13070179; doi:10.1177/23259582261431644)
Supplement: sj-docx-2-jia-10.1177_23259582261431644 - Supplemental material for mHealth Interventions to Promote HIV Self-Testing Among Key Populations: A Systematic Review of Effectiveness and Implementation Outcomes [file sj-docx-2-jia-10.1177_23259582261431644.docx]

# Effects of mHealth interventions on HIV self-testing among key populations: A systematic review

To enable PROSPERO to focus on COVID-19 submissions, this registration record has undergone basic automated checks for eligibility and is published exactly as submitted. PROSPERO has never provided peer review, and usual checking by the PROSPERO team does not endorse content. Therefore, automatically published records should be treated as any other PROSPERO registration. Further detail is provided [here.](https://www.crd.york.ac.uk/prospero/documents/PROSPEROLetterForAutoPublishJournalRejects.pdf)

# Citation

Review question

What are the effects of mHealth on HIV self-testing among key populations?

What mHealth interventions improve linkage to care following HIV self-testing among key populations?

# Searches

The following sources will be searched: PubMed/MEDLINE, Web of Science, CINAHL, Academic Search Complete, PsycArticles, PsycINFO, SocINDEX, CENTRAL, and grey literature (WHO, CDC, ECDC, UNICEF, UNAIDS, among others). We will include all published studies in the English language, starting in 2016 as this is the year WHO officially recommended HIV self-testing as a safe and effective way of testing for HIV.

# Types of study to be included

Inclusion criteria:

We will include several study designs such as randomized controlled trials, quasi-experimental studies, cohort studies, case-control, cross-sectional studies, mixed-method studies, qualitative studies, case studies, and evaluation reports.

Exclusion Criteria:

We will exclude protocols, letters, editorials, reviews, conference abstracts, and books.

# Condition or domain being studied

The effects of mobile health (mHealth) interventions strengthen HIV programs as they facilitate reaching underserved populations, improving treatment adherence, and simplifying HIV testing procedures. However, the effects of mHealth interventions on HIV self-testing among key populations remain unknown. While there is evidence about the impact of mHealth on HIV testing, little is known about the linkage to care following HIV self-testing among key populations. This review will synthesize the evidence on the effects of mHealth interventions on HIV self-testing uptake, acceptability, identification and prevalence of new HIV-positive diagnoses, and linkage to care among HIV self-testing users.

# Participants/population

Participants/population include key populations, such as female sex workers (FSW), men who have sex with men

(MSM), gay men, transgender people, people who inject drugs (PWID), heterosexuals, pregnant women, teenagers, and people in prisons.

# Intervention(s), exposure(s)

Inclusion criteria:

We will include any mobile-based interventions geared towards HIV self-testing, including but not limited to applications and information services. Telemedicine through mobile phones will also be included.

Exclusion criteria:

We will exclude technology-based interventions for HIV self-testing that do not involve mobile phones.

# Comparator(s)/control

The comparator of the study will be the usual care or standard care for HIV. This systematic review will also include studies on the intervention that do not contain a reference to the comparison group.

# Main outcome(s)

We will examine the effects of mHealth on HIV self-testing uptake, acceptability, and identification and prevalence of new HIV-positive diagnoses. We will also identify mHealth interventions facilitating linkage to care following HIV self- testing.

# Additional outcome(s)

We will include challenges focusing on barriers to implementing mHealth interventions for HIV self-testing and its recommendations.

# Data extraction (selection and coding)

We will be involved in the creation of the search strategy. Once we finalize the search strategy, we will conduct a search on the English databases, including grey literature. We will then conduct title and abstract screening of the studies obtained. After this, we will conduct a full-text screening of eligible articles. Any disagreements towards the inclusion of studies will be discussed within the team until a consensus is reached. If required, we will consult our supervisor for the final decision.

The data to be extracted include: title, citation (author, publication year, source), objectives, study location, study design, study setting, study population, sample size, types of mHealth, comparison group, reported outcomes, challenges, and recommendations.

# Risk of bias (quality) assessment

We will check and evaluate the risk for bias of included studies using specific tools designed for different study designs. We will employ the Cochrane Collaboration's risk of bias assessment tool for randomized controlled trials (RCTs), the ROBINS-I Tool for quasi-experimental studies, the National Institutes of Health (NIH) Quality Evaluation Tool for observational studies (e.g., cohort, case-control, cross-sectional studies), Critical Skills Appraisal Program Checklist for qualitative studies, and the Mixed-Method Appraisal Tool for mixed-method studies. For the certainty of evidence, we will use GRADE for quantitative studies and GRADE-CERQual for qualitative studies.

# Strategy for data synthesis

We will adhere to the PRISMA 2020 guidelines and flowchart to illustrate the results of our review. Given the broad

range of intervention strategies, target groups, and outcome measures for HIV self-testing, we will conduct a narrative synthesis by summarizing the quantitative and qualitative findings of individual studies. We will report descriptive statistics (e.g., percentages) and effect sizes (e.g., odds ratio, risk ratio) of quantitative studies. We will summarize the findings based on types of mHealth interventions, key populations, and geographic regions. We will conduct a thematic analysis for qualitative studies based on the reported themes and quotes. However, if enough RCTs with quality data have been gathered, we will conduct a meta-analysis of the effects of mHealth on HIV self-testing among key populations.

# Analysis of subgroups or subsets

If enough quality RCTs are available, we will conduct subgroup analysis based on types of mHealth interventions, key populations, and geographic regions.

Contact details for further information

Organisational affiliation of the review

Review team members and their organisational affiliations

Collaborators

Anticipated or actual start date

01 November 2023

# Anticipated completion date

01 April 2024

# Funding sources/sponsors

None

# Conflicts of interest

Language

English

| Country  Philippines |  |  |
| --- | --- | --- |
| Stage of review  Review Ongoing |  |  |
| Subject index terms status  Subject indexing assigned by CRD |  |  |
| Subject index terms  MeSH headings have not been applied to this record |  |  |
| Date of registration in PROSPERO  14 November 2023 |  |  |
| Date of first submission  03 November 2023 |  |  |
| Stage of review at time of this submission |  |  |
| **Stage** | **Started** | **Completed** |
| Preliminary searches | Yes | No |
| Piloting of the study selection process | No | No |
| Formal screening of search results against eligibility criteria | No | No |
| Data extraction | No | No |
| Risk of bias (quality) assessment | No | No |
| Data analysis | No | No |

*The record owner confirms that the information they have supplied for this submission is accurate and complete and they understand that deliberate provision of inaccurate information or omission of data may be construed as scientific misconduct.*

*The record owner confirms that they will update the status of the review when it is completed and will add publication details in due course.*

Versions

14 November 2023

14 November 2023
