# Supplementary material for: mHealth Interventions to Promote HIV Self-Testing Among Key Populations: A Systematic Review of Effectiveness and Implementation Outcomes
Source: J Int Assoc Provid AIDS Care. 2026 Apr 9;25:23259582261431644. doi: 10.1177/23259582261431644 (PMC13070179; doi:10.1177/23259582261431644)
Supplement: sj-pdf-3-jia-10.1177_23259582261431644 - Supplemental material for mHealth Interventions to Promote HIV Self-Testing Among Key Populations: A Systematic Review of Effectiveness and Implementation Outcomes [file sj-pdf-3-jia-10.1177_23259582261431644.pdf]

Supplementary File 3. Search Strategy

Strategy 1: Search terms for "key population"
key population [tw]
OR transgender [MeSH] OR transgender [tw]
OR multiple sexual partner [MeSH] OR multiple sexual partner [tw]
OR gay men [tw]
OR men who have sex with men [tw]
OR MSM [tw]
OR heterosexual [MeSH] OR heterosexual [tw]
OR sex workers [MeSH] OR sex workers [tw]
OR prostitutes [tw]
OR female sex workers [tw]
OR client of sexually transmitted infection [tw]
OR people who inject drugs [tw]
OR PWID [tw]
OR intravenous drug user [tw]
OR IV drug user [tw]
OR pregnant women [MeSH] OR pregnant women [tw]
OR teenagers [tw]
OR adolescent [MeSH] OR adolescent [tw]
OR people in prison [tw]

Strategy 2: Search terms for "mHealth"
mHealth [tw]
OR mobile health [tw]
OR mobile phone [tw]
OR mobile devices [tw]
OR mobile technology [tw]
OR cellular phone [tw]
OR cellphone [MeSH] OR cellphone [tw]
OR smartphone [MeSH] OR smartphone [tw]
OR SMS [tw]
OR mobile information services [tw] OR information services [MeSH] OR information services [tw]
OR text messaging [MeSH] OR text messaging [tw] OR text [tw]
OR mobile applications [MeSH] OR mobile applications [tw] OR applications [tw] OR apps [tw] OR phone calls [tw]
OR telemedicine [MeSH] OR telemedicine [tw]

Strategy 3: Search terms for "HIV self-testing"
HIV self-testing [tw] OR HIVST [tw]
OR uptake [tw] OR utilization [tw] OR completion [tw]
OR acceptability [tw] OR acceptance rate [tw]
OR identification of people living with HIV [tw]
OR yield of new HIV-positive diagnoses [tw]
OR prevalence of new HIV-positive diagnosis [tw]
OR linkage to care following HIV self-testing [tw] OR linkage to care [tw] OR linkage to treatment [tw]

Search period: December 1, 2016 – January 1, 2024

Database	Initial search	After removing duplicates
PubMed/MEDLINE	2,124	2,118
Web of Science	3,681	3,676
CINAHL	9,622	9,401
Academic Search Premier	2,150	2,111
PsycArticles	141	141
PsycINFO	246	194
SocINDEX	334	125
CENTRAL	6,911	6,507
**TOTAL**	**25,209**	**24,273**

936 duplicates removed

(1) Pubmed/MEDLINE: 2,124 articles

((key population [tw] OR transgender [MeSH] OR transgender [tw] OR multiple sexual partner [MeSH] OR multiple sexual partner [tw] OR gay men [tw] OR men who have sex with men [tw] OR MSM [tw] OR heterosexual [MeSH] OR heterosexual [tw] OR sex workers [MeSH] OR sex workers [tw] OR prostitutes [tw] OR female sex workers [tw] OR client of sexually transmitted infection [tw] OR people who inject drugs [tw] OR PWID [tw] OR intravenous drug user [tw] OR IV drug user [tw] OR pregnant women [MeSH] OR pregnant women [tw] OR teenagers [tw] OR adolescent [MeSH] OR adolescent [tw] OR people in prison [tw]) AND (mHealth [tw] OR mobile health [tw] OR mobile phone [tw] OR mobile devices [tw] OR mobile technology [tw] OR cellular phone [tw] OR cellphone [MeSH] OR cellphone [tw] OR smartphone [MeSH] OR smartphone [tw] OR SMS [tw] OR mobile information services [tw] OR information services [MeSH] OR information services [tw] OR text messaging [MeSH] OR text messaging [tw] OR text [tw] OR mobile applications [MeSH] OR mobile applications [tw] OR applications [tw] OR apps [tw] OR phone calls [tw] OR telemedicine [MeSH] OR telemedicine [tw])) AND (HIV self-testing [tw] OR HIVST [tw] OR uptake [tw] OR utilization [tw] OR completion [tw] OR acceptability [tw] OR acceptance rate [tw] OR identification of people living with HIV [tw] OR yield of new HIV-positive diagnoses [tw] OR prevalence of new HIV-positive diagnosis [tw] OR linkage to care following HIV self-testing [tw] OR linkage to care [tw] OR linkage to treatment [tw]) Filters: Full text, English, from 2016/12/1 - 2024/01/01

(2) Web of Science: 3,681 articles

((TS=(key population OR transgender OR multiple sexual partner OR gay men OR men who have sex with men OR MSM OR heterosexual OR sex workers OR prostitutes OR female sex workers OR client of sexually transmitted infection OR people who inject drugs OR PWID OR intravenous drug user OR IV drug user OR pregnant women OR teenagers OR adolescent OR people in prison)) AND TS=(mHealth OR mobile health OR mobile phone OR mobile devices OR mobile technology OR cellular phone OR cellphone OR smartphone OR SMS OR mobile information services OR information services OR text messaging OR text OR mobile applications OR applications OR apps OR phone calls OR telemedicine)) AND TS=(HIV self-testing OR HIVST OR uptake OR utilization OR completion OR acceptability OR acceptance rate OR identification of people living with HIV OR yield of new HIV-positive diagnoses OR prevalence of new HIV-positive diagnosis OR linkage to care following HIV self-testing OR linkage to care OR linkage to treatment) and Article (Document Types) and English (Languages) and 2024 or 2023 or 2022 or 2021 or 2020 or 2019 or 2018 or 2017 or 2016 (Publication Years) and BIOSIS Citation Index or Zoological Record (Database)

(3) CINAHL: 9,622 articles

"TX (key population OR transgender OR multiple sexual partner OR gay men OR men who have sex with men OR MSM OR heterosexual OR sex workers OR prostitutes OR female sex workers OR client of sexually transmitted infection OR people who inject drugs OR PWID OR intravenous drug user OR IV drug user OR pregnant women OR teenagers OR adolescent OR people in prison) AND TX (mHealth OR mobile health OR mobile phone OR mobile devices OR mobile technology OR cellular phone OR cellphone OR smartphone OR SMS OR mobile information services OR information services OR text messaging OR text OR mobile applications OR applications OR apps OR phone calls OR telemedicine) AND TX (HIV self-testing OR HIVST OR uptake OR utilization OR completion OR acceptability OR acceptance rate OR identification of people living with HIV OR yield of new HIV-positive diagnoses OR prevalence of new HIV-positive diagnosis OR linkage to care following HIV self-testing OR linkage to care OR linkage to treatment) Full Text; Publication Date: 20161201-20240101; English Language; Peer Reviewed; Research Article; Exclude Pre-CINAHL; Exclude MEDLINE records; Publication Type: Journal Article; PDF Full Text; Language: English AND Apply equivalent subjects on 2024-01-01 03:06 AM"

(4) Academic Search Premier: 2,150 articles

"TX (key population OR transgender OR multiple sexual partner OR gay men OR men who have sex with men OR MSM OR heterosexual OR sex workers OR prostitutes OR female sex workers OR client of sexually transmitted infection OR people who inject drugs OR PWID OR intravenous drug user OR IV drug user OR pregnant women OR teenagers OR adolescent OR people in prison) AND TX (mHealth OR mobile health OR mobile phone OR mobile devices OR mobile technology OR cellular phone OR cellphone OR smartphone OR SMS OR mobile information services OR information services OR text messaging OR text OR mobile applications OR applications OR apps OR phone calls OR telemedicine) AND TX (HIV self-testing OR HIVST OR uptake OR utilization OR completion OR acceptability OR acceptance rate OR identification of people living with HIV OR yield of new HIV-positive diagnoses OR prevalence of new HIV-positive diagnosis OR linkage to care following HIV self-testing OR linkage to care OR linkage to treatment) Full Text; Peer Reviewed; Published Date: 20161201-20240101; Document Type: Article; Language: English; PDF Full Text AND Apply equivalent subjects on 2024-01-01 02:55 AM"

(5) PsycArticles: 141 articles

"TX (key population OR transgender OR multiple sexual partner OR gay men OR men who have sex with men OR MSM OR heterosexual OR sex workers OR prostitutes OR female sex workers OR client of sexually transmitted infection OR people who inject drugs OR PWID OR intravenous drug user OR IV drug user OR pregnant women OR teenagers OR adolescent OR people in prison) AND TX (mHealth OR mobile health OR mobile phone OR mobile devices OR mobile technology OR cellular phone OR cellphone OR smartphone OR SMS OR mobile information services OR information services OR text messaging OR text OR mobile applications OR applications OR apps OR phone calls OR telemedicine) AND TX (HIV self-testing OR HIVST OR uptake OR utilization OR completion OR acceptability OR acceptance rate OR identification of people living with HIV OR yield of new HIV-positive diagnoses OR prevalence of new HIV-positive diagnosis OR linkage to care following HIV self-testing OR linkage to care OR linkage to treatment) Full Text; Publication Year: 2016-2024; Publication Date: 20161101-20240101; Peer Reviewed; Publication Status: Fully Published; Document Type: Journal Article AND Apply equivalent subjects on 2024-01-01 04:00 AM"

(6) PsycINFO: 246 articles

"TX (key population OR transgender OR multiple sexual partner OR gay men OR men who have sex with men OR MSM OR heterosexual OR sex workers OR prostitutes OR female sex workers OR client of sexually transmitted infection OR people who inject drugs OR PWID OR intravenous drug user OR IV drug user OR pregnant women OR teenagers OR adolescent OR people in prison) AND TX (mHealth OR mobile health OR mobile phone OR mobile devices OR mobile technology OR cellular phone OR cellphone OR smartphone OR SMS OR mobile information services OR information services OR text messaging OR text OR mobile applications OR applications OR apps OR phone calls OR telemedicine) AND TX (HIV self-testing OR HIVST OR uptake OR utilization OR completion OR acceptability OR acceptance rate OR identification of people living with HIV OR yield of new HIV-positive diagnoses OR prevalence of new HIV-positive diagnosis OR linkage to care following HIV self-testing OR linkage to care OR linkage to treatment) Linked Full Text; Publication Date: 20161201-20240101; Peer Reviewed; Publication Status: fully published; Publication Type: Peer Reviewed Journal; English language; Language: English AND Apply equivalent subjects on 2024-01-01 04:11 AM"

(7) SocINDEX: 334 articles

"TX (key population OR transgender OR multiple sexual partner OR gay men OR men who have sex with men OR MSM OR heterosexual OR sex workers OR prostitutes OR female sex workers OR client of sexually transmitted infection OR people who inject drugs OR PWID OR intravenous drug user OR IV drug user OR pregnant women OR teenagers OR adolescent OR people in prison) AND TX (mHealth OR mobile health OR mobile phone OR mobile devices OR mobile technology OR cellular phone OR cellphone OR smartphone OR SMS OR mobile information services OR information services OR text messaging OR text OR mobile applications OR applications OR apps OR phone calls OR telemedicine) AND TX (HIV self-testing OR HIVST OR uptake OR utilization OR completion OR acceptability OR acceptance rate OR identification of people living with HIV OR yield of new HIV-positive diagnoses OR prevalence of new HIV-positive diagnosis OR linkage to care following HIV self-testing OR linkage to

care OR linkage to treatment) Full Text; Peer Reviewed; Publication Date: 20161201-20240101; Document Type: Article; Language: English AND Apply equivalent subjects on 2024-01-01 04:18 AM"

(8) CENTRAL: 6,911 articles

(key population OR transgender OR multiple sexual partner OR gay men OR men who have sex with men OR MSM OR heterosexual OR sex workers OR prostitutes OR female sex workers OR client of sexually transmitted infection OR people who inject drugs OR PWID OR intravenous drug user OR IV drug user OR pregnant women OR teenagers OR adolescent OR people in prison) AND (mHealth OR mobile health OR mobile phone OR mobile devices OR mobile technology OR cellular phone OR cellphone OR smartphone OR SMS OR mobile information services OR information services OR text messaging OR text OR mobile applications OR applications OR apps OR phone calls OR telemedicine) AND (HIV self-testing OR HIVST OR uptake OR utilization OR completion OR acceptability OR acceptance rate OR identification of people living with HIV OR yield of new HIV-positive diagnoses OR prevalence of new HIV-positive diagnosis OR linkage to care following HIV self-testing OR linkage to care OR linkage to treatment) (Word variations have been searched)" with Publication Year from 2016 to 2024, with Cochrane Library publication date Between Dec 2016 and Jan 2024, in Trials (Word variations have been searched)
