# Supplementary material for: mHealth Interventions to Promote HIV Self-Testing Among Key Populations: A Systematic Review of Effectiveness and Implementation Outcomes
Source: J Int Assoc Provid AIDS Care. 2026 Apr 9;25:23259582261431644. doi: 10.1177/23259582261431644 (PMC13070179; doi:10.1177/23259582261431644)
Supplement: sj-pdf-4-jia-10.1177_23259582261431644 - Supplemental material for mHealth Interventions to Promote HIV Self-Testing Among Key Populations: A Systematic Review of Effectiveness and Implementation Outcomes [file sj-pdf-4-jia-10.1177_23259582261431644.pdf]

Supplementary File 4. Extended PICO

Study, year (country)	Study design	Study setting	Study population	Sample size	mHealth intervention	Comparator	Reported outcomes
Balán, 2022 (United States)	Mixed methods	New York City between November 2016 and September 2017.	HIV-uninfected, non-monogamous MSM and TGW, aged 18 or older, reporting at least three occasions of condomless anal intercourse over the past three months, and rarely or never using condoms during anal intercourse	48	SMARTtest smartphone app	No comparison group	37 out of 48 participants (77%) have used the INSTI to self-test (M=3.7 times; SD=3.9). 100% of participants stated that they would use INSTI for self-testing. The app has an average functionality rating of 4.16 out of 5 (SD=0.85) and an average helpfulness rating of 6.12 out of 7 (SD=1.09). 78% of the participants would also recommend the app to a friend.
Biello, 2021 (United States)	Mixed Methods-RCT	LYNX, Chicago, IL (CORECenter) and Tampa, FL (University of South Florida); and for MyChoices, Boston, MA (Fenway Health), the Bronx, NY (Children's Hospital at Montefiore), Chapel Hill, NC (University of North Carolina at Chapel Hill).	Cisgender men aged 15-24 years who had not undergone HIV testing in the past three months, self-reported as HIV-uninfected or of unknown HIV status, owned an iOS or Android mobile phone capable of downloading. the LYNX or MyChoices app, were. fluent in English, not using PrEP, had. not received an experimental HIV vaccine, were not enrolled in another. HIV intervention study and had no health conditions that could affect study participation or outcomes, with self-reported evidence of being at high. risk for HIV acquisition	80	LYNX and MyChoices app	Standard care	65% of participants ordered an HIVST kit during the study while 75% of those who ordered reported using at least one self-test kit over the study period. 87% reported that it was extremely/very helpful to be able to order these kits through the apps. It was noted that the convenience of HIVST and privacy satisfaction led to high acceptability. Moreover, 80% reported. that in the future use, it would be very convenient. to use the test correctly using the apps.

Chan, 2021 (China)	Mixed methods	Hongkong	Hong Kong Chinese-speaking males aged ≥18 years old who have had anal. sex with at least one man in the last six months are willing to provide contact. information for post-test evaluation, have access to live chat applications like Line, Skype, or Facetime, and are not already HIV-infected	350	HIVST Online via phone/social media	No comparison group	40.4% of new users received HIVST-online during the project period while 63.1% of ever-users received HIVST-online. The overall HIV testing rates led to 79.4% of ever-users and 58.6% of newm users who received any form of HIV testing during the period. More importantly, 4 HIVST-online users were screened to be HIV positive and have been linked to treatment and care successfully.
De Boni, 2019 (Brazil)	Cross-sectional	Between February 2015 and January 2016 in Curitiba, Southern Brazil. In partnership with the Department of STI, AIDS, and Viral Hepatitis of the Brazilian Ministry of Health; the Municipal Health Secretariat of Curitiba; the Federal University of Paraná; Grupo Dignidade (a local Lesbian Gay Bisexual Transgender and Queer/Questioning NGO), and Centers for Disease Control	MSM, aged 18 years or older, residing in Curitiba, Brazil, who were either HIV-negative or unaware of their HIV status	3,218	Internet-based platform through mobile and online technologies	No comparison group	17,786 individuals have visited the website while 3,218 of them completed the online questionnaires. 2,526 HIVST packages were delivered. 71.7% reported that it was very easy to use the platform while 19.6% stated it was easy. 93.6% found that the testing instructions were clear.

		and Prevention (CDC)/Brazil.					
Drake, 2020 (Kenya)	Cohort	Kisumu County, Kenya.	HIV-negative women aged 14 and older	2,123	mSurvey SMS system	Traditional in-person method	Out of 2,123 participants, 486 accepted HIVSTs, with 359 eligible for mSurvey, of which 222 registered. Among these, 76 had follow-ups with 62 reporting outcomes, while 146 without follow-ups had 87 reporting. Of the 222 registered, 96/102 recommended HIVSTs via SMS, 92/96 reported partner usage, and 17/102. experienced negative partner reactions reported. through SMS.
Gous, 2020 (South Africa)	Cross-sectional	Inner-city Johannesburg, South Africa. Hillbrow Health Clinic	18 years or older	300	AspectTM HIV-self-testing mobile app	No comparison group	A significant majority of participants (98.7%) found the app easy to use, although 8.7% encountered difficulties. 99.7% of participantsm stated that they were willing to use the app again
Huang, 2016 (United States)	Cross-sectional	West Hollywood and downtown Los Angeles.	Black or Latino men who have sex. with men (MSM), aged 18 years or older	122	GrindrTM	No comparison group	In the follow-up survey, 96.5% of respondents reported that the self-tests were easy or very easy. to use, and while 96.5% tested HIV negative, the two individuals who tested positive promptly. sought further medical care. Additionally, 68.4% preferred self-testing over other methods, highlighting the method's effectiveness and acceptance.

Kelvin, 2019 (Kenya)	RCT	Eight roadside wellness clinics in Kenya run by the North Star Alliance.	Female sex workers registered in the North Star Alliance electronic health record system who was not known to be HIV-positive, resided in Kenya, had. a valid mobile phone number, had fewer than four HIV tests in the past 12 months, and had not had an HIV. test in the past three months	2,196	Text messages	Enhanced Standard of Care and Traditional Standard of Care	During a 2-month follow-up, the intervention group, which was notified about the availability. of HIV self-testing kits via text, saw a significant increase in HIV testing rates (10.8%) compared. to the enhanced SOC group that received general testing reminders (6.1%). Within the intervention group, 119 female sex workers chose to test, with 59.7% opting for self-test kits—72.2% of these. tests were supervised in clinics, while 27.8% took kits for home use
Larsson, 2023 (Zimbabwe)	Mixed methods - RCT	Three provinces (Harare,Bulawayo, and Mashonaland East)	16–24 years in Zimbabwe who had not been tested for HIV in the past 12 months and were not known to be HIV.positive	2,181	ITHAKA	Provider-delivered HIV testing	Among 2,181 youth accessing HIV testing, only 128.(5.9%) opted for HIV self-testing (HIVST) using the ITHAKA app. Of these, 108 of the 109 (99.1%) participants who used the app on-site completed them testing with all reporting nonreactive results. However, only 9 out of 19 (47.4%) who used the app. off-site finished their testing journey, with most. results non-reactive except for one participant who reported a reactive result in person at the CHIEDZA center.
Lin, 2023 (China)	Cluster RCT	Selected 10 cities from Shandong	Biologically male, aged 18 years or older, residing in one	935	WeChat	Standard HIV	The study found higher self-reported HIV testing uptake

		Province, China, including Jinan, Weifang, Zibo, Jining, Liaocheng, Qingdao, Dezhou, Weihai, Binzhou, and city Zaozhuang	of the 11 specified cities in Shandong province, HIV seronegative or of unknown status, had engaged in anal sex with a man in the past 12 months, and had not been tested for HIV in the past 3 months			prevention	in the intervention group compared to the control at 3, 6, 9, and 12 months, with significant increases noted at the 6-, 9-, and 12-month follow-ups. Cluster-level analysis showed an 11.62% increase in testing rates in intervention cities (P=.04), demonstrating community-wide impact. However, the individual-level effect indicated a 69% higher odds of testing in the intervention arm, but this was not statistically significant (risk ratio 1.69, 95% CI 0.87-3.27; P=.11), indicating variability in individual responses.
Logie, 2021 (Uganda)	Cluster RCT	Between 2020 and 2021, across five informal settlements. (Kabalagala, Kansanga, Katwe, Nsambya and Rubaga)	16–24 years, living in specific informal settlements in Kampala, Uganda, who spoke one of the study languages (English, French, Swahili, Luganda, Kinyarwanda, or Kirundi) and had access to a mobile phone	450	Bidirectional supportive SMS	Standard of Care	Results indicated substantial increases in HIV testing uptake, with the HIVST arm increasing from 27.6% at baseline to 91.2% at 8 months, and the HIVST + mHealth arm from 30.9% to 94.2%. Additionally, at 12 months, correct HIV status knowledge was significantly higher in the intervention arms (100% in HIVST and 97.9% in HIVST + mHealth) compared to 61.5% in the standard of care (SOC) arm. Secondary outcomes showed modest changes, including varying impacts on depression and stigma across the groups, with notable increases in

							HIV-related stigma in the HIVST arm and reductions in adolescent SRH stigma in the HIVST + mHealth arm.
Mancuso, 2023 (United States)	RCT	Atlanta, Detroit and New York City.	HIV-negative men who have sex with men (MSM)	417	M-cubed mobile app	Wait-listed control group	Over half of the participants (53%) in the intervention arm of the M-cubed trial ordered an HIV self-test kit, with those planning to test within the next 3 months and those not tested in the past 3 months being significantly more likely to order one, regardless of their income, race, or age.
Marley, 2021 (China)	Cross-sectional 18 years or older, who had previously tested for HIV with a human	Jiangsu province in the eastern part of China	Male individuals at birth, aged 16 years or older, who had engaged in sexual activities with other men, resided in Jiangsu province, and were willing to provide informed consent	692	Smartphone based electronic readers	Traditional methods	The study revealed that 71.2% (493 out of 692) of the participants expressed a willingness to use smartphone-based electronic readers (SERs) for HIV self-testing. Among those who were willing, 98% indicated that the use of SERs would likely lead them to self-test for HIV more frequently.
Ntinga, 2022 (South Africa)	Cross-sectional 18 years or older, who had previously tested for HIV with a human	KwaZulu-Natal, South Africa.	18 years or older, who had previously tested for HIV with a human counselor, resided in the Lindela or neighboring communities, could use a smartphone to interact with the Nolwazi_bot	120	Nolwazi_bot	Traditional human counselor-led HIV testing experience	The study revealed strong acceptance and preference for the chatbot compared to traditional human counselors, with 79.2% of participants favoring the chatbot experience. The chatbot was seen as engaging in a human-like manner by 77.5% of participants, attributed to its realistic style of interaction. Additionally, approximately 17.5% of participants tested

							positive for HIV and were directed to receive further medical care.
Pai, 2021 (South Africa)	Quasi-experimental	Cape Town, South Africa, between January 2017 and June 2018.	18 years or older, of unknown HIV status within the past three months, with access to an Android/iPhone smartphone or the capability to use a tablet/smartphone for HIVST, excluding those already on antiretroviral therapy (ART), with a confirmed HIV diagnosis, or with a serious medical condition requiring hospitalization	2,262	HIVSmart	Conventional HIV testing	The study in South Africa evaluated an app-based HIV self-testing (HIVST) program against conventional HIV testing (ConvHT). Results showed that the HIVST program, which includes both supervised and unsupervised options via the app, achieved nearly perfect linkage rates to follow-up care, with 99.7% for unsupervised and 99.8% for supervised testing, compared to 98.5% for ConvHT. Additionally, the HIVST program detected more new HIV infections (9% overall) and significantly increased test referrals (16.7%), demonstrating the app's effectiveness in enhancing outreach and engagement compared to the conventional method (6.79% infections detected, 3.1% referrals). The app-based program effectively leveraged digital connectivity to improve HIV testing outcomes.
Rosengren, 2016 (United States)	Cross-sectional	Los Angeles County; West Hollywood and Central Los Angeles.	Black or Latino men who have sex with men (MSM), aged 18 years or older, residing in high HIV incidence areas	4,389	GrindrTM	No comparison group	There was notable engagement with 4,389 unique visitors and 333 requests for kits over four weeks. The majority opted for mail delivery (247 requests),

							while others chose pharmacy vouchers (58 requests) or vending machine pickup (28 requests). Follow-up responses from 56 users showed that all administered their tests, with two testing positive for HIV and seeking further medical care. This initiative underscores the potential of mobile apps as effective tools in public health strategies to engage populations less likely to use traditional testing methods.
Shrestha, 2023 (Malaysia)	Mixed methods	University of Malaya, Kuala Lumpur, Malaysia,	Cis-gender men aged 18 years or older, self-reporting as HIV-negative or unknown status, PrEP-naive, at risk for HIV acquisition according to WHO guidelines, owning a smartphone	50	JomPrEP	No comparison group	The JomPrEP study showed high effectiveness and acceptability of a smartphone app designed to facilitate HIV prevention among men who have sex with men (MSM) in Malaysia. Notably, 84% of participants used the app to order HIV self-testing kits, with 42% ordering more than once during the 30-day study period. The app was highly accepted, achieving a mean System Usability Scale (SUS) score of 73.8 (SD = 10.1), significantly above the acceptability threshold of 50. These results underscore the app's role in providing private, convenient access to HIV testing and prevention services, effectively addressing significant barriers

							faced by the target population.
Wu, 2021 (Southern China)	Cohort	Zhuhai, southern China.	Men aged 16 years or older, born biologically male, who had ever engaged in sexual activity with another man	371	WeChat	No comparison group	In the study on social media-based HIV/syphilis self-testing among Chinese MSM, the mHealth approach via WeChat led to a 99% return rate of test kits, with 1141 out of 1150 kits returned and 1099 valid results. This platform significantly enhanced participant engagement, demonstrated by 40% of alters being naive HIV testers compared to 21% of indexes, showcasing the effectiveness of mobile technology in boosting compliance and reach in public health interventions.
Zhang, 2021 (China)	RCT	4 major Chinese cities (Beijing, Shenyang, Chongqing, and Shenzhen) from December 2018 to September 2019.	MSM aged 18-65 years who engaged in high-risk sexual behaviors, tested negative on a fourth-generation HIV test, and showed no severe liver or kidney dysfunction	939	WeChat	Standard care	Through WeChat, participants received their HIV test kits, instructions, and could consult with healthcare providers in real-time. They also used the app to submit their test results. This mobile health (mHealth) approach led to high participation rates: at a three-month check-in, about 74.5% of participants had used their HIV test kits. Furthermore, over half of these users shared kits with others, and around 52.8%% used the kits with their partners right before or after sexual activity. Using WeChat simplified the testing process, improved

							convenience, and maintained privacy, which are all crucial for encouraging frequent testing and engagement in a community that values discretion and support from peers.
Zhao, 2018 (China)	Qualitative	Hefei, Anhui Province, China.	18 years or older, residing in Hefei, who had condomless anal sex with at least one male partner in the past six months, owned a smartphone capable of using WeChat, and were HIV-negative or of unknown status	36	WeChat	No comparison group	Key outcomes include the acceptance of WeChat for information dissemination if privacy concerns are addressed through secure and discreet communication strategies. Participants preferred official accounts managed by credible sources to enhance trust and recommended careful management of message content, frequency, and timing to respect privacy and ensure engagement. The study highlighted the importance of integrating user feedback and maintaining cultural and context-specific relevance in the design of mHealth interventions.
Zhu, 2019 (China)	RCT	September 2017 to June 2018 in Hefei, the capital city of Anhui Province, China.	Cis-gender male MSM, aged 18 or older, with a history of unprotected anal sex in the past six months, HIV-negative or status unknown, currently residing in Hefei without plans to move, willing to undergo HIV self-testing, and possessing a smartphone capable of using WeChat	100	WeTest, a private WeChat group	Control group that received only two oral HIV self-testing kits, without the additional WeChat-based support and	The intervention group showed significantly higher rates of HIV testing (adjusted rate ratio [RR] = 1.99, 95% confidence interval [CI] = 1.07–3.84) and specifically, a greater use of oral HIV self-testing kits (adjusted RR = 2.17, 95% CI = 1.08–4.37) compared to the control group. Additionally,

						information	regardless of which group participants were in, there was a notable increase over time in the rates of consistent condom use with both main partners (adjusted RR = 18.13, 95% CI = 5.19–63.31) and non-main partners (adjusted RR = 5.33, 95% CI = 2.35–12.08). This suggests an overall improvement in safe sexual practices among all participants throughout the study.
