# Supplementary material for: mHealth Interventions to Promote HIV Self-Testing Among Key Populations: A Systematic Review of Effectiveness and Implementation Outcomes
Source: J Int Assoc Provid AIDS Care. 2026 Apr 9;25:23259582261431644. doi: 10.1177/23259582261431644 (PMC13070179; doi:10.1177/23259582261431644)
Supplement: sj-pdf-6-jia-10.1177_23259582261431644 - Supplemental material for mHealth Interventions to Promote HIV Self-Testing Among Key Populations: A Systematic Review of Effectiveness and Implementation Outcomes [file sj-pdf-6-jia-10.1177_23259582261431644.pdf]

Supplementary File 6. Excluded studies with reason

No.	Study ID	Reason for exclusion
1	Adeagbo, 2019	Not related to mHealth
2	Akinola, 2021	Not related to mHealth
3	Altaf, 2023	Not related to mHealth
4	Balán, 2020	In its development phase
5	Baraitser, 2019	Not related to mHealth
6	Bell, 2019	Not related to mHealth
7	Chen, 2023	Unfinished study
8	Chiu, 2016	Outcomes not related
9	Conserve, 2019	Unfinished study
10	Dana, 2019	Outcomes not related
11	Dana, 2023	Outcomes not related
12	De Boni, 2018	Insufficient study
13	den Daas, 2020	Not related to mHealth
14	Elorreaga, 2022	Not related to mHealth
15	Fischer, 2021	Population criteria not related
16	Fischer, 2023	Not related to mHealth
17	Gabriel, 2018	Not related to mHealth
18	Gavin, 2023	Population criteria not related
19	Hoagland, 2021	Not related to HIVST
20	Jantarapakde, 2018	Not related to mHealth
21	Jin, 2020	Not related to mHealth
22	Kelvin, 2019	Population criteria not related
23	Kumwenda, 2023	Outcomes not related
24	Kutner, 2021	Outcomes not related
25	Kwan, 2022	Outcomes not related
26	Kwan, 2023	Not related to mHealth
27	Lemley, 2020	Unpublished study
28	Lightfoot, 2018	Not related to mHealth
29	Lippman, 2023	Not related to HIVST
30	Logie, 2021	Not related to mHealth
31	Logie, 2022	Not related to mHealth
32	Logie, 2023	Not related to mHealth
33	Lora, 2020	Not related to mHealth
34	Lu, 2020	Not related to mHealth
35	Lu, 2021	Outcomes not related
36	MacGowan, 2020	Not related to mHealth
37	Martínez-Riveros, 2023	Not related to mHealth
38	Muwanguzi, 2021	Not related to mHealth
39	Nct, 2016	Trial protocol
40	Nct, 2019	Trial protocol
41	Nct, 2022	Trial protocol
42	Nct, 2022	Trial protocol
43	Nicholls, 2022	Not related to mHealth

44	Phanuphak, 2018	Not related to mHealth
45	Ronen, 2018	Not related to HIVST
46	Senn, 2017	Not related to HIVST
47	Sheira, 2022	Not related to mHealth
48	Sibanda, 2018	Conference abstract
49	Trujillo, 2020	Not related to mHealth
50	Uhrig Castonguay, 2020	Not related to mHealth
51	Venter, 2019	Not related to mHealth
52	Witzel, 2019	Not related to mHealth
53	Witzel, 2020	Not related to mHealth
54	Witzel, 2020	Not related to mHealth
55	Wray, 2020	Unfinished study
56	Xiu, 2022	Not related to mHealth
57	Zhou, 2023	Not related to mHealth
