# Supplementary material for: mHealth Interventions to Promote HIV Self-Testing Among Key Populations: A Systematic Review of Effectiveness and Implementation Outcomes
Source: J Int Assoc Provid AIDS Care. 2026 Apr 9;25:23259582261431644. doi: 10.1177/23259582261431644 (PMC13070179; doi:10.1177/23259582261431644)
Supplement: sj-pdf-7-jia-10.1177_23259582261431644 - Supplemental material for mHealth Interventions to Promote HIV Self-Testing Among Key Populations: A Systematic Review of Effectiveness and Implementation Outcomes [file sj-pdf-7-jia-10.1177_23259582261431644.pdf]

Supplementary File 7. Risk of bias of observational studies

Risk of bias assessment of quasi-experimental studies
(Please indicate whether low, moderate, serious, critical, no information)

Author, Year	Selection of participants	Confounding Variables	Class of Intervention	Deviation from Intended Intervention	Missing Data	Measurement of the outcomes	Selection of the Reported Results	Overall risk of bias
Pai, 2021	Low	Low	Low	Moderate	Moderate	Low	Low	Moderate

Risk of bias assessment of observational cohort and cross-sectional studies
(Please indicate whether yes, no, CD [cannot determine], NA [not applicable], NR [not reported])

Author, Year	1	2	3	4	5	6	7	8	9	10	11	12	13	14	Overall Rating
De Boni, 2019	Yes	Yes	Yes	Yes	No	Yes	Yes	No	Yes	Yes	Yes	No	No	No	Fair
Drake, 2020	Yes	Yes	No	Yes	No	Yes	Yes	No	Yes	Yes	Yes	No	No	NR	Fair
Marley, 2021	Yes	Yes	Yes	Yes	No	Yes	No	No	Yes	No	Yes	No	NR	Yes	Fair
Ntinga, 2022	Yes	Yes	Yes	Yes	No	Yes	Yes	No	Yes	No	Yes	No	NR	No	Fair
Wu ,2021	Yes	Yes	Yes	Yes	No	Yes	Yes	No	Yes	Yes	Yes	NR	NR	NR	Good
Gous ,2020	Yes	Yes	Yes	Yes	No	Yes	NR	No	Yes	No	Yes	NR	NR	NR	Fair
Rosengren,2016	Yes	Yes	Yes	Yes	No	Yes	NR	Yes	Yes	No	Yes	NR	NR	NR	Fair
Huang,2014	Yes	Yes	Yes	Yes	No	Yes	Yes	No	Yes	Yes	Yes	NR	Yes	NR	Good

Was the research question or objective in this paper clearly stated? 2: Was the study population clearly specified and defined? 3: Was the participation rate of eligible persons at least 50%? 4: Were all thesubjects selected or recruited from the same or similar populations (including the same time period)? Were inclusion and exclusion criteria for being in the study prespecified and applied uniformly to all participants? 5: Was a sample size 1. Is there an adequate rationale for using a mixed methods design to address the research question? 2. Are the different components of the study effectively integrated to answer the research question? 3. Are the outputs of the integrated qualitative and quantitative components adequately interpreted? 4. Are divergences and inconsistencies between quantitative and qualitative results adequately addressed? 5. Do the different components of the study adhere to the quality criteria of each tradition of the methods involved? 6. Is the qualitative approach appropriate to answer the research question? 7. Are the qualitative data collection methods adequate to address the research question? 8. Are the findings adequately derived from the data? 9. Is the interpretation of results sufficiently substantiated by data? 10. Is there coherence between qualitative data sources, collection, analysis, and interpretation? 11. Is randomization appropriately performed? 12. Are the groups comparable at baseline? 13. Are there complete outcome data? 14. Are outcome assessors blinded to the intervention provided? 15, Did the participants adhere to the assigned intervention? justification, power description, or variance and effect estimates provided? 6: For the analyses in this paper, were the exposure(s) of interest measured prior to the outcome(s) being measured? 7: Was the timeframe sufficient so that one could reasonably expect to see an association between exposure and outcome if it existed? 8: For exposures that can vary in amount or level, did the study examinedifferent levels of the exposure as related to the outcome (e.g., categories of exposure, or exposure measured as a continuous variable)? 9: Were the exposure measures (independent variables) clearlydefined, valid, reliable, and implemented consistently across all study participants? 10: Was the exposure(s) assessed more than once over time? 11: Were the outcome measures (dependent variables) clearly defined, valid, reliable, and implemented consistently across all study participants? 12 Were the outcome assessors blinded to the exposure status of participants? 13: Was the loss to follow-up after baseline 20% or less? 14: Were key potential confounding variables measured and adjusted statistically for their impact on the relationship between exposure(s) and outcome(s)?

Risk of bias assessment of qualitative studies
(Please indicate whether yes, no, or can't tell)

Author, Year	1	2	3	4	5	6	7	8	9	10	Overall Rating
Zhao, 2018	Yes	Yes	Yes	Yes	Yes	Yes	Yes	Yes	Yes	Yes	Good

1: Was there a clear statement of the aims of the research? 2: Is a qualitative methodology appropriate? 3: Was the research design appropriate to address the aims of the research? 4: Was the recruitment strategy appropriate to the aims of the research? 5: Was the data collected in a way that addressed the research issue? 6: Has the relationship between the researcher and participants been adequately considered? 7: Have ethical issues been taken into consideration? 8: Was the data analysis sufficiently rigorous? 9: Is there a clear statement of findings? 10: Is the research valuable?

Risk of bias assessment of mixed methods studies
(Please indicate whether yes, no, or can't tell)

Author, Year	1	2	3	4	5	6	7	8	9	10	11	12	13	14	15	Overall Rating
Balán, 2022	Yes	Yes	Yes	Yes	Yes	Yes	Yes	Yes	Yes	Yes	Yes	Yes	Yes	No	Yes	Fair
Biello, 2021	Yes	Yes	Yes	Yes	No	Yes	Yes	Yes	Yes	Yes	Yes	Yes	Yes	Yes	Yes	Good
Larsson, 2023	Yes	Yes	Yes	No	Yes	Yes	Yes	Yes	Yes	Yes	Yes	Can't T	Yes	No	No	Good
Shrestha, 2023	Yes	Yes	Yes	No	Yes	Yes	Yes	Yes	Yes	Yes	Yes	Yes	Yes	No	Yes	Fair
Chan, 2021	Yes	Yes	Yes	Yes	Yes	Yes	Yes	Yes	Yes	Yes	Yes	Yes	Yes	Yes	Yes	Good

1. Is there an adequate rationale for using a mixed methods design to address the research question? 2. Are the different components of the study effectively integrated to answer the research question? 3. Are the outputs of the integrated qualitative and quantitative components adequately interpreted? 4. Are divergences and inconsistencies between quantitative and qualitative results adequately addressed? 5. Do the different components of the study adhere to the quality criteria of each tradition of the methods involved? 6. Is the qualitative approach appropriate to answer the research question? 7. Are the qualitative data collection methods adequate to address the research question? 8. Are the findings adequately derived from the data? 9. Is the interpretation of results sufficiently substantiated by data? 10. Is there coherence between qualitative data sources, collection, analysis, and interpretation? 11. Is randomization appropriately performed? 12. Are the groups comparable at baseline? 13. Are there complete outcome data? 14. Are outcome assessors blinded to the intervention provided? 15 . Did the participants adhere to the assigned intervention?
