# Supplementary material for: mHealth Interventions to Promote HIV Self-Testing Among Key Populations: A Systematic Review of Effectiveness and Implementation Outcomes
Source: J Int Assoc Provid AIDS Care. 2026 Apr 9;25:23259582261431644. doi: 10.1177/23259582261431644 (PMC13070179; doi:10.1177/23259582261431644)
Supplement: sj-pdf-8-jia-10.1177_23259582261431644 - Supplemental material for mHealth Interventions to Promote HIV Self-Testing Among Key Populations: A Systematic Review of Effectiveness and Implementation Outcomes [file sj-pdf-8-jia-10.1177_23259582261431644.pdf]

Supplementary File 8. GRADE Summary of findings

Intervention: Use of mHealth
Comparison: Standard Care

Outcomes	Impact	No. of Participants (Studies)	Quality of Evidence
**Likelihood of Use**	Fourteen studies assessed the effects of mHealth on the likelihood of HIVST (HIV self-testing) use. Thirteen studies found a positive effect, while one study found no impact	4,176 (4 RCT; 3 Mixed method; 1 Cluster Randomized Trial)	⊕⊕○○ LOW
**Willingness of Continue of HIVST use**	Five studies demonstrate a strong willingness to continue using HIVST facilitated by various mHealth services, all showing positive effects and no mixed or insignificant findings	50 (1 Mixed method)	⊕⊕○○ LOW
**HIVST Acceptability**	Three studies have demonstrated positive effects, with no mixed findings and no significant negative effects	471 (1 RCT)	⊕⊕○○ LOW
**Satisfaction with using HIVST**	One study shows satisfaction with HIV	350 (1 Mixed	

	self-testing (HIVST) use and positive effects, with no mixed or insignificant findings	method)	⊕⊕○○<br>LOW
**Linkage to Care**	One study demonstrates high linkage to care among participants using HIV self-testing (HIVST) and positive effects, with no mixed or insignificant findings	2262 (1 Quasi Experimental)	⊕○○○<br>VERY LOW
**Barriers to implementing mHealth interventions for HIVST and its recommendation**	A total of 16 studies have reported various barriers in implementing mHealth interventions for HIVST. It showed no effect at all and has no mixed findings and no significant negative effects	5824 (4RCT; 3 Mixed method; 1 Quasi Experimental)	⊕⊕○○<br>LOW

GRADE Working Group grades of Evidence:

High Quality: We are very confident that the true effect lies close to that of the estimate of the effect.
Moderate Quality: We are moderately confident in the effect estimate: The true effect is likely to be close to the estimate of the effect, but there is a possibility that it is substantially different.
Low Quality: Our confidence in the effect estimate is limited: The true effect may be substantially different from the estimate of the effect.
Very Low Quality: We have very little confidence in the effect estimate: The true effect is likely to be substantially different from the estimate of effects.
