# Supplementary material for: mHealth Interventions to Promote HIV Self-Testing Among Key Populations: A Systematic Review of Effectiveness and Implementation Outcomes
Source: J Int Assoc Provid AIDS Care. 2026 Apr 9;25:23259582261431644. doi: 10.1177/23259582261431644 (PMC13070179; doi:10.1177/23259582261431644)
Supplement: sj-pdf-9-jia-10.1177_23259582261431644 - Supplemental material for mHealth Interventions to Promote HIV Self-Testing Among Key Populations: A Systematic Review of Effectiveness and Implementation Outcomes [file sj-pdf-9-jia-10.1177_23259582261431644.pdf]

Supplementary File 9. GRADE CERQual

Key Findings	Studies contributing to the review finding	Assessment of methodological limitations	Assessment of relevance to the research question	Assessment of coherence	Assessment of adequacy	Overall CERQual assessment of confidence	Explanation of judgment
**High acceptability of application use**	Balan,2022<br>De Boni, 2019<br>Ntinga, 2022<br>Biello, 2021<br>Chan,2021	**Moderate methodological limitations.**<br><br>Average CASP rating: 12.0<br><br>Average MMAT rating: 14.3<br><br>Insufficient rationale was provided for the chosen study design and data analysis methods.	**Minor concerns about relevance**<br><br>The findings were related to the research question.	**Minor concerns about coherence**<br><br>The data was consistent across the studies.	**Moderate concerns about adequacy**<br><br>Limited richness and quantity of data and participants.	**Moderate**	The primary issue of concern relates to the adequacy of evidence supporting the key finding.
**Likelihood of use of HIVST**	Balan,2022<br>Gous, 2020<br>Huang,2021<br>Wu, 2021<br>Rosengren, 2016<br>Biello, 2021<br>Shrestha, 2023<br>Zhao,2018<br>Larsson, 2023	**Moderate methodological limitations.**<br><br>Average CASP rating: 10.5<br><br>Average MMAT rating: 13.2	**Moderate concerns about relevance**<br><br>Findings on the likelihood of use of HIVST were at times not related to the main research question.	**Moderate concerns about coherence**.<br><br>Three studies showed unclear results.	**Moderate concerns about adequacy**<br><br>Limited richness and quantity of data	**Low**	The primary issue of concern relates to the adequacy of evidence supporting the key finding.

Key Findings	Studies contributing to the review finding	Assessment of methodological limitations	Assessment of relevance to the research question	Assessment of coherence	Assessment of adequacy	Overall CERQual assessment of confidence	Explanation of judgment
**HIVST acceptability**	Gous,2020<br>Rosengen,2016<br>Drake, 2020	**Moderate methodological limitations.**<br><br>Average CASP rating: 7.3<br><br>Insufficient rationale was provided for the chosen study design and data analysis methods.	**Major concerns about relevance**<br><br>Findings on HIVST acceptability were at times not related to the main research question.	**Moderate concerns about coherence**<br><br>Two studies showed mixed results and only one study showed a positive impact on HIVST acceptability.	**Major concerns about adequacy**<br><br>Limited richness and quantity of data	**Moderate**	The primary issue of concern relates to the adequacy of evidence supporting the key finding.
**Willingness of continuation of HIVST use**	Gous, 2020<br>Marley,2021<br>Wu, 2021<br>Shrestha, 2023<br>Zhao, 2018	**Moderate methodological limitations.**<br><br>Average CASP rating: 9.4<br><br>Average MMAT rating: 13.0<br><br>Insufficient rationale was provided for the chosen study design and data analysis methods.	**Minor concerns about relevance**<br><br>The findings were related to the research question.	**Moderate concerns about coherence**<br><br>Data from three studies were consistent across the studies and two had unclear results.	**Moderate concerns about adequacy**<br><br>Limited richness and quantity of data	**Low**	The primary issue of concern relates to the adequacy of evidence supporting the key finding.

Key Findings	Studies contributing to the review finding	Assessment of methodological limitations	Assessment of relevance to the research question	Assessment of coherence	Assessment of adequacy	Overall CERQual assessment of confidence	Explanation of judgment
Satisfaction with using HIVST	Chan,2021	**Moderate methodological limitations.**<br><br>Average CASP rating: 15.0<br><br>Average MMAT rating: 15.0<br><br>Insufficient rationale was provided for the chosen study design and data analysis methods.	**Minor concerns about relevance.**<br><br>Findings were related to the research question	**Moderate concerns about coherence.**<br><br>Illustrative quotes are missing in the text.	**Major concerns about adequacy**<br><br>Only one study and offers thin data.	**Low**	The primary issue of concern relates to the adequacy of evidence supporting the key finding.

CASP — Critical Appraisal Skills Programme, MMAT — Mixed Methods Appraisal Tool
